# An evolutionary approach to recover genes predominantly expressed in the testes of the zebrafish, chicken and mouse

**DOI:** 10.1186/s12862-019-1462-8

**Published:** 2019-07-03

**Authors:** Sophie Fouchécourt, Floriane Picolo, Sébastien Elis, Charlotte Lécureuil, Aurore Thélie, Marina Govoroun, Mégane Brégeon, Pascal Papillier, Jean-Jacques Lareyre, Philippe Monget

**Affiliations:** 1 PRC, CNRS, IFCE, INRA, Université de Tours, 37380 Nouzilly, France; 2 Institut de Recherche sur la Biologie de l’Insecte (IRBI), UMR 7261, CNRS-Université de Tours, 37200 Tours, France; 3 INRA, UPR 1037, Laboratory of Fish Physiology and Genomics (LPGP), BIOSIT, OUEST-genopole, Bât. 16, Campus de Beaulieu, cedex, 35042 Rennes, France

**Keywords:** Gene evolution, Conservation, Testis, Spermatogenesis

## Abstract

**Background:**

Previously, we have demonstrated that genes involved in ovarian function are highly conserved throughout evolution. In this study, we aimed to document the conservation of genes involved in spermatogenesis from flies to vertebrates and their expression profiles in vertebrates.

**Results:**

We retrieved 379 *Drosophila melanogaster* genes that are functionally involved in male reproduction according to their mutant phenotypes and listed their vertebrate orthologs. 83% of the fly genes have at least one vertebrate ortholog for a total of 625 mouse orthologs. This conservation percentage is almost twice as high as the 42% rate for the whole fly genome and is similar to that previously found for genes preferentially expressed in ovaries. Of the 625 mouse orthologs, we selected 68 mouse genes of interest, 42 of which exhibited a predominant relative expression in testes and 26 were their paralogs. These 68 mouse genes exhibited 144 and 60 orthologs in chicken and zebrafish, respectively, gathered in 28 groups of paralogs. Almost two thirds of the chicken orthologs and half of the zebrafish orthologs exhibited a relative expression ≥50% in testis. Finally, our focus on functional in silico data demonstrated that most of these genes were involved in the germ cell process, primarily in structure elaboration/maintenance and in acid nucleic metabolism.

**Conclusion:**

Our work confirms that the genes involved in germ cell development are highly conserved across evolution in vertebrates and invertebrates and display a high rate of conservation of preferential testicular expression among vertebrates. Among the genes highlighted in this study, three mouse genes (*Lrrc46, Pabpc6* and *Pkd2l1*) have not previously been described in the testes, neither their zebrafish nor chicken orthologs. The phylogenetic approach developed in this study finally allows considering new testicular genes for further fundamental studies in vertebrates, including model species (mouse and zebrafish).

**Electronic supplementary material:**

The online version of this article (10.1186/s12862-019-1462-8) contains supplementary material, which is available to authorized users.

## Background

Cells of the germline and molecular processes that result in the production of high-quality gametes are of particular importance for animal sexual reproduction. Spermatogenesis can be divided into three main stages: mitotic, meiotic and spermiogenic stages. The formation of spermatozoa suitable for fertilisation represents a complex process that requires the expression of numerous genes [1]. Nearly 2000 different genes can be involved in testicular development, germ cell differentiation, meiosis and the successive stages of spermiogenesis [2]. Nevertheless, the gene networks and regulatory pathways involved in spermatogenesis have yet to be fully identified, and achieving this objective may facilitate an understanding of the causes of infertility generated intrinsically by genetic defects and/or induced by extrinsic environmental perturbations.

There are high similarities between bird and mammal testis physiology and regulation: the organisation of the seminiferous tubules is similar, with the various differentiation stages of germ cells (spermatogonia, spermatocytes, spermatids) being surrounded by the Sertoli cells, which provide the microenvironment for their proliferation and differentiation. Likewise, the histology of the interstitial tissue that includes Leydig cells producing androgens is similar [3]. In contrast to mammals, the avian testes are located within the body cavity. Consequently, spermatogenesis occurs at body temperature, whereas it occurs 5–8 °C cooler than the body temperature in mammals [4]. Another difference is that spermatogenesis in birds is more rapid (2 weeks in chickens, ducks and turkeys) compared with mammals (2 months in humans) [5] [6]. In chickens, peak fertility is reached within the first 6–10 months of life [7] [8].

Given that spermatogenesis represents a relatively well-conserved process even among phylogenetically distant animal species, it is likely that certain underlying genetic mechanisms are conserved during evolution. Indeed, evolutionary distant animal species share most mechanisms involved in basic processes of germ cell production [9, 10]. The conservation of spermatogenic regulators can be illustrated by the well-known *Boule* gene, whose loss of function induces spermatogenetic arrest and, ultimately, azoospermia in fruit flies and vertebrates. Interestingly, the phenotype can be rescued in these mutants by the human *BOULE* gene [11]. Nonetheless, although involved in basic processes, species- or clade-specific genes have been identified: for instance, *asterless* (*asl*) is required for male meiosis in fruit flies, but this gene is not maintained across vertebrate evolution [12]. Previous works on testicular gene evolution have deciphered the orthology link between a small number of genes and/or between a limited number of species. Some species are only partially documented relative to model species such as the fruit fly (*Drosophila melanogaster*), mouse (*Mus musculus*) and zebrafish (*Danio rerio*). Among vertebrates, chickens lack substantial data concerning testis molecular regulation, although they are somewhat considered a model species (owing to the availability of genomic data). Another interesting and singular point concerning the chicken is that this species is of high agronomic interest. The identification of reliable fertility markers in farm chickens is critical in the context of the assessment of reproductive performance. Indeed, genetic selection (based on growth performance) in this species has led to unexpected and deleterious consequences for reproductive performance, including a reduction in the longevity of male reproduction and a deterioration in sperm quality [13, 14].

In the present study, we propose a comparative biological approach combining phenotypic, phylogenetic and expression profile analyses in invertebrate and vertebrate species, based on our previous work on ovarian genes [15]. Here, we aimed 1) to obtain an overview of testicular gene conservation between invertebrates (fruit fly) and vertebrates (mouse, chicken, zebrafish); 2) to obtain an overview of the expression profiles of conserved testicular genes in vertebrates and 3) to propose new (i.e. not yet described) candidate genes required for spermatogenesis, particularly in chickens. The two strengths of the study can be seen in the exploitation of 1) in silico data in three model species, including one invertebrate (the fruit fly) and two vertebrates (the mouse and the zebrafish), and 2) a large set of genes (almost 400). Our investigation demonstrates the conservation of genes involved in the determination, maintenance and/or differentiation of germ cells. Moreover, this study offers a substantial list of uncharacterized genes for chicken testis function, which might be of agronomic interest regarding the need for fertility markers in avian species.

## Results

**Fig. 1:**
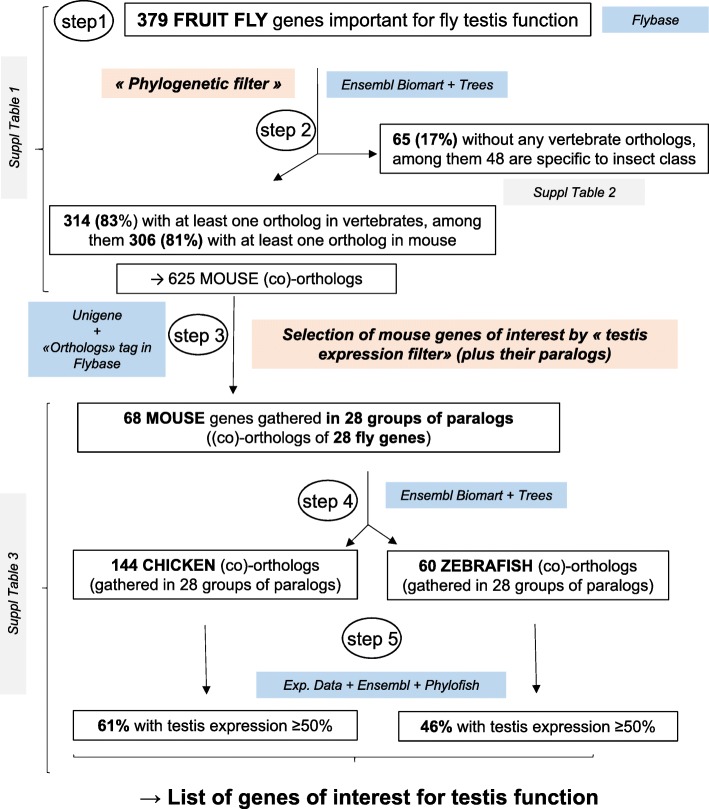
Workflow and results, with numbers and percentages of genes of testis interest in the species studied. Step 1: selection of **379 fruit fly genes** with functional interest for testis. Step 2: retrieval of their orthologs (“phylogenetic filter”) in vertebrates, including mice, for a total of 625 mouse orthologues. Step 3: to tighten the list, selection of genes with a high relative testis expression (“preferential testis expression filter”) and their paralogs (scored in Flybase for their orthology link) to obtain a final list of **68 mouse genes** of interest, gathered in 28 groups of paralogs. Step 4: identification of chicken and zebrafish orthologs (144 and 60, respectively) of these mouse genes, and finally, the determination of their levels of relative testis expression (fifth step). Genes conserved across evolution exhibiting an arbitrary level of rTE ≥ 50% in a species were considered genes of interest for testis function in this species.

Figure 1 summarizes the workflow of our study with its main steps and results. This workflow aims to 1) evaluate the conservation of testis genes between invertebrates and vertebrates, and to 2) evaluate whether a gene that is highly expressed in mouse testes is also highly expressed in chicken and/or zebrafish testes. In reference to Fig. 1, step 1 was to select 379 genes with a functional interest for fly testis (see M&M section 1.1). Step 2 comprised the retrieval (“phylogenetic filter”) of their orthologs in vertebrates (including mice) and various invertebrates (see M&M section 1.2). Subsequently, to establish a list of mouse genes of interest to testis function, step 3 was to select those mouse orthologs (from those obtained in step 2) that exhibited a predominant relative testicular expression (see M&M section 1.3), as well as their paralogs (see M&M section 1.4). Next, the zebrafish and chicken orthologs of these mouse genes were retrieved (step 4; see M&M section 1.2), along with information regarding their testicular expression profile (step 5; see M&M section 1.5). Finally, GO and mutant phenotypes (when available) of the genes of interest were analysed (see M&M sections 1.6 and 1.7).

### Evolution of genes whose mutation gives rise to a disturbed male phenotype in fruit flies (Fig. 1 steps 1 and 2)

**Fig. 2:**
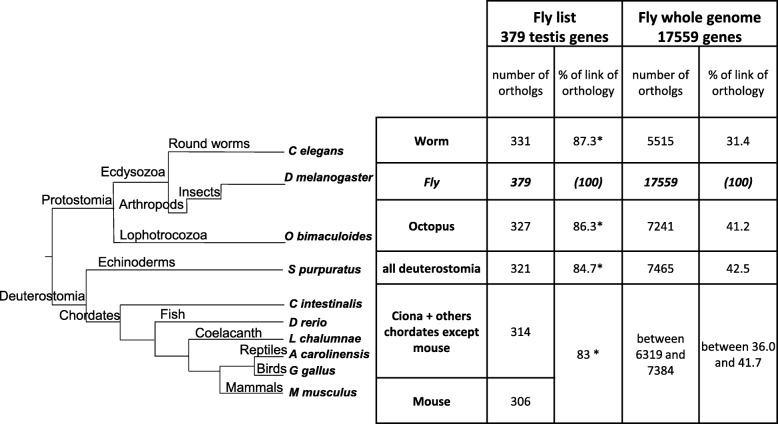
Schematic evolution of the 379 fruit fly genes in a simplified tree of life. The number of orthologs of the 379 fly genes was obtained for various species using Ensembl metazoa gene and Ensembl gene databases (release 88), as described in Material and Methods. The percentage of conservation was compared to the entire gene repertoire using the Chi-square test. * indicates a significant difference (*p* < 0.0001)

We listed **379 fly genes** (identity information presented in columns B-I, Additional file 1: Table S1) for which a mutation gives rise to a male defective reproductive phenotype in this species according to Flybase -Dmel Release 6.13- (Fig. 1 step 1). For 181 of these genes (48%), the mutation also has a phenotypic effect in females (column B). Figure 2 summarises the presence of the 379 fruit fly genes in various invertebrate and vertebrate genomes in the tree of life (using Biomart Ensembl release 88). Among the 379 fly genes, 306 (81%) possess at least one ortholog in mice, for a total of 625 mouse co-orthologs (Fig. 1 step 2; columns J-M in Additional file 1: Table S1). Among the 73 remaining genes without mouse orthologs, we sought (co)-orthologs in five other species (chosen as model or informative species), with the objective of estimating whether these genes can be found in other chordates: yellow sea-squirt (*Ciona intestinalis)*, coelacanth (*Latimeria chalumnae),* zebrafish (*Danio rerio*), anole lizard (*Anolis carolinensis*) and chicken (*Gallus gallus*). We identified eight fly genes (Jyalpha, Moe, Ntl, Sxl, Syx13, topi, Vps28 and w) with at least one ortholog in at least one of these species. In total, at least **83%** (314 = 306 + 8 of 379) of the fruit fly genes are conserved in vertebrates. This is almost twice the rate (42%) for the whole genome (*p* < 0.0001). No vertebrate ortholog was found for the 65 remaining genes (i.e. **17%** of the list, Figs. 1 and 2, and Additional file 2: Table S2). The clades where these genes are present were retrieved from the *Ensembl* (release 88) trees when available (20 genes highlighted in blue in Additional file 1: Table S1, Additional file 2: Table S2) or, when unavailable, from the *EnsemblMetazoa* trees (45 genes highlighted in orange in Additional file 1: Table S1, Additional file 2: Table S2). Among these 65 genes with no ortholog in chordates, only seven genes (11%) are found in both protostomes and the Echinoderm clade, whereas 58 (89%) are specifically found in protostomes, with 48 (74%) being specific to insects (columns G and N, Additional file 2: Table S2).

### Tissue expression of mouse orthologs and retrieval of their chicken and zebrafish orthologs

We subsequently retrieved vertebrate (co)-orthologs of the 379 fly genes and focused on those expressed in testes in the species of interest (mouse, chicken, zebrafish). Thus, the objective was 1) to establish a tightened list of mouse genes exhibiting a predominant testicular expression profile (Fig. 1, step 3); 2) to retrieve the orthologs of these mouse genes in two other vertebrates, namely the zebrafish as a fish model species and the chicken as an avian model species (Fig. 1, step 4) and 3) to determine whether these (co)-orthologs are also expressed in the testes of these two species (Fig. 1, step 5) and if their relative testis expression (rTE) is also predominant. These co-orthologs are listed in Additional file 3: Table S3. The thresholds for designating a predominant testis-expressed gene were arbitrarily chosen as follows: in mouse, these are genes with rTE ≥ 50%, or between 20 and 50% if they are expressed in less than five tissues, whereas these are genes with rTE ≥ 50% in chicken and zebrafish.

### List of mouse genes of interest for the testes among the 625 orthologs (Fig. 1, step 3)

For each of the **625 mouse orthologs**, we estimated their relative tissue expression levels using data from the Unigene “EST profile” database. This database has proved highly reliable in predicting the expression profiles of reproductive genes in mice ([16] [17] [18] [19]), even though, to be rigourous, we verified gene expression by qRT-PCR in this species. The calculated (in silico-predicted) rTE varies from 0 to 100% (column BK in Additional file 1: Table S1). As in our previous work on ovarian genes, we arbitrarily decided to focus on genes whose relative testicular expression was above a certain threshold, chosen here either ≥50% (34 orthologs) or between 20 and 50% (eight orthologs) and with expression restricted in two to five tissues among the 45 adult tissues listed in the Unigene EST profile (total: 42 orthologs among 625). We retrieved complemental information concerning the orthology link between fly and mouse co-orthologs: paralogs (26 genes) of these 42 mouse genes predominantly expressed in testes were considered when they exhibited a noteworthy orthology link according to Flybase (score –available in Flybase Dmel 6.26- equal or higher than the corresponding ortholog gene; see M&M section 1.4). We finally identified **68 mouse genes** of interest (42 orthologs + 26 paralogs), listed in Additional file 3: Table S3 (columns G-L). These mouse genes are co-orthologs of **28 fruit fly genes** (columns B-F) and are gathered in **28 groups of paralogs** with at least one gene preferentially expressed in the testes. We also determined if fly and mouse orthologs belong to the same tree or superTREE (recently available in Ensembl release 96, column K). Among the genes with an rTE ≥ 20%, 13 are exclusively expressed in the testes according to Unigene (calculated rTE = 100%).

Twenty of the 68 mouse genes were arbitrarily chosen to verify their testis-enriched or exclusive expression by qRT-PCR analyses. We thus experimentally confirmed the Unigene profile for each of them (Additional file 4: Figure S1).

### Retrieval of chicken and zebrafish orthologs of the 68 mouse genes of testis interest (Fig. 1, step 4)

The chicken and zebrafish orthologs of the 68 mouse genes of testis interest (or 28 groups of mouse paralogs) were identified using the Biomart tool and analysis of phylogenetic trees and syntenic chromosomal fragments (Fig. 1, step 4; Ensembl release 88).

**→ Chicken orthologs** (Additional file 3: Table S3A, ID information in columns M/N): each of the 28 groups of mouse paralogs possesses at least one chicken ortholog. Among the 68 mouse genes, four have no orthologs in chicken (*RhoA, Shcbp1l, Lmna* and *Lmntd1).* The 28 groups of mouse paralogs total **144 chicken (co-)orthologs**, most of which being localised on conserved syntenic genomic regions (columns Q/R). The details of the orthology relation are described in Additional file 3: Table S3B.

**→ Zebrafish orthologs** (Additional file 3: Table S3A, ID information columns S/T): among the 28 groups of mouse paralogs, 26 possess at least one zebrafish ortholog. The details of the orthology relation are described in Additional file 3: Table S3B. In total, 10 mouse genes have no zebrafish orthologs: *Shcbp1l* (but its paralog *Shcbp1* has one) and *Lmntd1* as observed in chicken, *plus Boll, Lrrc46, Dmrtb1, Myh15, Phf7, Tcp10a, Tcp10b, Tcp10c*. The 26 groups of mouse paralogs corresponded to **60 zebrafish (co)-orthologs.** The difference in the number of orthologs for the 68 mouse genes in chickens and zebrafish (144 and 60, respectively) is primarily due to the fact that mouse *Sun3/Sun5/Spag4* paralogs and *Phf7* have one or no ortholog in zebrafish, compared to 31 and 63 in chickens, respectively. Among the 60 zebrafish orthologs, half of the genes (31) are localised on conserved syntenic chromosomal fragments in comparison with mouse (columns U/V).

### Relative testis expression of the chicken and zebrafish orthologs for identification of genes of interest for testis function (Fig. 1 step 5)

**Fig. 3:**
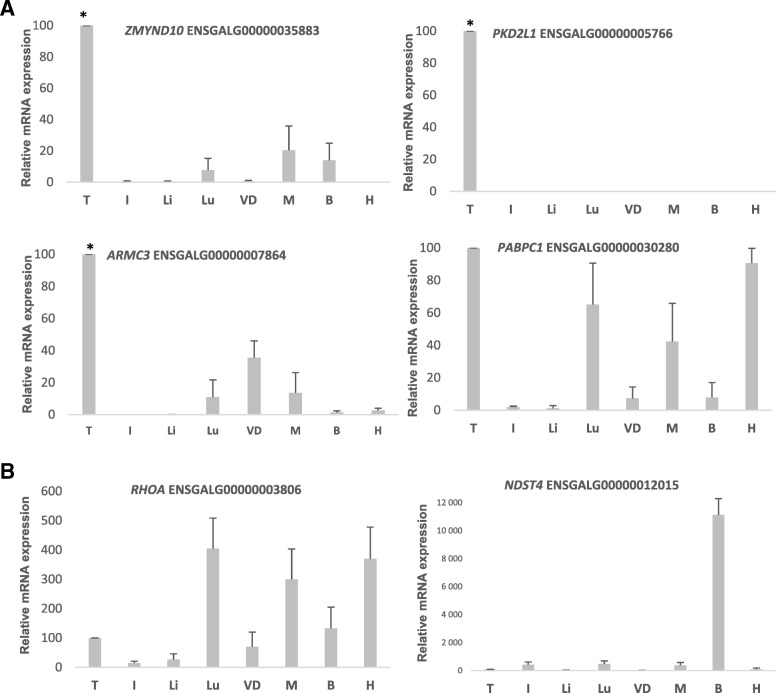
Relative mRNA expression level (mean +/− SEM) in chicken tissues for six representative genes investigated by qRT-PCR (see other genes in Additional file 5: Figure S2). These genes (A) present a preferential relative expression in testes (70% for *ZMYND10*, 61% for *ARMC3*, 30% for *PABPC1, 100% for PKD2L1*) or (B) are ubiquitous (*RHOA*) or highly expressed in another organ (*NDST4* in the brain). Tissues are (*N* = 3 different adult males): T: testis; I: intestine; Li: liver; Lu: lung; VD: vas deferens; M: muscle; B: brain; H: heart. The testis level is arbitrarily equal to 100, and normalisation was performed with the *EEF1A* housekeeping gene. The specific primers are described in Additional file 7: Table S5. The relative mRNA testis expression levels are indicated in Additional file 3: Table S3A, column O (as percentages). ANOVA, followed by Bonferroni *post-hoc* tests, was used to compare tissular expression in chickens (* means *p* < 0.001)

We then aimed to identify, among the genes described above, those putatively exhibiting an enriched testicular expression in chickens and/or zebrafish. Given the lack of previous studies regarding chickens, we determined most expression data by qRT-PCR (Fig. 3, Additional file 5: Figure S2 and Additional file 3: Table S3A, column O). We also used Ensembl (chicken) and Phylofish (zebrafish) databases to retrieve expression data (Additional file 3: Table S3A, column P for chickens and column W for zebrafish). We arbitrarily deemed that the expression of a gene is enriched in the testes when its rTE is ≥50%. To better compare with the mouse, we also considered genes with their rTE between 20 and 50%.

**→ Testis expression of chicken orthologs** (Additional file 3: Table S3A, columns O -our results- and P –Ensembl data-): among the 144 chicken co-orthologs of the 68 mouse genes, we retrieved data for 143 genes (no data available for *HOXC12*): **106** genes (74%) including the numerous co-orthologs of *Sun5/Spag4* and *Phf7 (*20 and 63 duplicated gene copies respectively) have their rTE ≥ 50%. Five genes in addition to the 11 *Sun3*-co-orthologs have their rTE between 20 and 50%.

Finally, among the 28 groups of mouse paralogs with at least one gene preferentially expressed in the testis, **17 (61%) have at least one chicken ortholog with a relative testis expression ≥ 50%** and four with an expression between 20 and 50%.

**→ Testis expression of zebrafish orthologs (**Additional file 3: Table S3A, column W): among the 60 zebrafish (co)-orthologs of the 68 mouse genes, 18 (30%) exhibit an rTE ≥ 50% in testis and/or ovary. For 20 (33%), the rTE values are between 20 and 50%.

Finally, among the 28 groups of mouse paralogs, **13 (46%) have at least one zebrafish ortholog with a relative testicular expression ≥ 50%**, and 10 with an expression between 20 and 50%.

The results above are summarised in Fig. 1, with ortholog numbers and percentages at each step of the workflow. The chicken and zebrafish genes with an rTE ≥ 50% are underlined in Additional file 3: Table S3A (columns N and T, respectively).

### Function(s) of testis genes conserved across evolution

To obtain an overview of the function of the genes emerging from the present study, we report data concerning their function, when available, in the fruit fly (Flybase data), mouse (MGI data) and zebrafish (review of the literature) (Additional file 3: Table S3A, columns E-L-X for fruit fly, mouse and zebrafish, respectively). Note that no functional data are currently available for chickens. In a second step, we also analysed enriched gene ontologies in the fly and mouse.

**In the case of the fruit fly**, of the 28 genes that have at least one testis-enriched mouse ortholog, almost all present a germ cell defect when mutated, independent of their testis expression (note that only 13 have a testis-enriched profile). For example, for *Zmynd10*, *TTLL3B* and *sqd*, the phenotype mutant is manifested in spermatozoon, spermatid axoneme and spermatogonium, respectively (see Additional file 3: Table S3A, column C). **In the zebrafish**, of these 60 orthologs, morpholino-injected mutant phenotypes are available for only five, with four exhibiting a defect only in males (female phenotype for *hnrnpdl*): *tdrd, tdr6a, dazl and rhoab*. For the four genes, the defect concerns an alteration of the germplasm structure. **In the mouse**, 15 genes exhibit germ cell defect mutants, with consecutive male infertility in almost all cases, whereas for the other genes, the data are “normal reproductive phenotype” (three genes), “no phenotype data available” (20 genes), “no reproductive phenotype described” (29 genes), as well as one exhibiting an embryonic lethality. Finally, for the three species, most keywords describing the testicular defects are related to germ cell development.

**Fig. 4:**
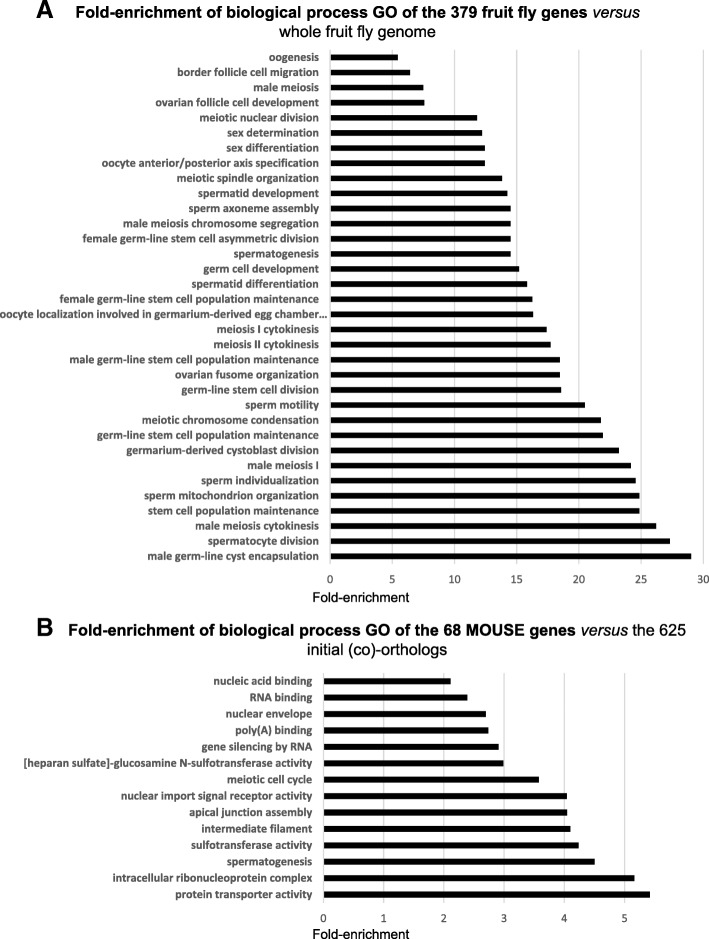
**a** Thirty-four “reproduction-related” biological process GOs enriched in the 379 fly genes compared to the whole genome (*p* < 0.01), according to the DAVID Functional Annotation Tool (see Additional file 6: Table S4); Fold enrichment arbitrarily chosen > × 5. **b** Comparison between GOs of the 625 and 68 mouse orthologs (fold enrichment arbitrarily chosen > × 2; Chi-square test, p < 0.01).

We also listed the GO of the candidate genes, analysed with the DAVID and Biomart tool (see M&M). When compared with the whole genome, the initial list of the 379 fruit fly genes exhibits 79 enriched GO terms (*p* < 0.01) with a fold-enrichment > × 3 (Additional file 6: Table S4); among them, almost half (34) are related to male/female gonad- or germ cell-functions and development (Fig. 4a), with a fold enrichment > × 5, including “GO:0007283 spermatogenesis” (× 14.5). This GO term is also significantly (p < 0.01) enriched for the 625 mouse orthologs (versus whole genome), with a 2.79-fold enrichment (not shown). In the mouse, comparison between the GO terms of the 625 orthologs and the restricted list of 68 genes predominantly expressed in the testes resulted in 14 enriched GO terms (fold enrichment > × 2, p < 0.01), including “spermatogenesis” (fold enrichment > × 4) and four notable functions related to nucleic acid binding (GO:0031047 “gene silencing by RNA”, GO:0008143 “poly(A) binding”, GO:0003723 “RNA binding”, GO:0003676 “nucleic acid binding”) (Fig. 4b). In summary, the genes can be classified into four main biological functions: meiosis process, interaction with DNA, RNA processing, binding or transport, structure of spermatid/spermatozoon.

## Discussion

In this study, we aimed to provide an overview of the genomic and expressional conservation of testis genes across evolution. The main results are: 1) testis genes are highly (83%) conserved between vertebrates and invertebrates; 2) of those conserved genes that are enriched in mouse testes, 61 and 46% presented a relative testis expression ≥50% in chickens and zebrafish, respectively; 3) most functions of these genes are related to germ cell development.

### Conservation of testis genes across evolution

The high rate of conservation (83%) found for testis genes between invertebrates and vertebrates is significantly higher than the 42% expected for the complete fruit fly genome and similar to that found for genes involved in ovarian function in our previous study (78%) [15]. This high rate is in accordance with the conservation of germ cell differentiation [1]. Moreover, this significantly high percentage is not singular or restricted to germ cell genes, as it is common for genes involved in drastic phenotypes, such as in our study. Indeed, Catillo-Davis & Harlt (2003) point out that genes whose mutation leads to a non-viable phenotype in invertebrates (*C. elegans*) are more conserved in eukaryotes than others [20]. Maxwell et al. (2014) also show that genes involved in human diseases exhibit a significantly higher conservation rate (52%) than would be expected for a random subset of all human genes [21].

We found that few mouse genes predominantly expressed in the testes have no chicken and/or zebrafish orthologs (no chicken ortholog: *RhoA, Shcbp1l, Lmndt1* and *Lmna*; no zebrafish ortholog: *Boll*, *Lrrc46, Dmrtb1, Shcbp1l, Lmndt1, Myh15, Phf7, Tcp10a/b/c*). This result is surprising for at least four genes absent in zebrafish (*Boll*, *Lrrc46, Dmrtb1* and *Phf7)*, as these genes are known to be important in fly and/or mouse testes (see references in paragraph *Conservation of testicular gene functions*) and are enriched in chicken testes (present results). Moreover, *Lrrc46, Dmrtb1* and *Boll* have orthologs in other cartilaginous and bony fish species, (data not shown). This discrepancy between zebrafish and other teleostean fish species has previously been observed for other genes involved in reproductive function. For instance, no gene encoding for the GnRH1 neuropeptide has been identified in zebrafish, although this hypophysiotropic GnRH1 form is essential to the release of LH in other fish species [22, 23]. Similarly, the *stra8* gene, which is required for the meiotic transition of spermatogonia in mammals, has not been identified in the zebrafish genome, while it is present in other fish genomes [24]. It has been proposed that the loss of evolutionary conserved genes in zebrafish strains is a result of the domestication of this species [25].

Synteny investigation, which is necessary to validate gene losses, is arduous in chicken and zebrafish for different reasons (incomplete annotation in chicken and supplemental round of whole genome duplications in teleosts). Moreover, updating the data bank with genomic data is an ongoing process. Thus, it may be possible that orthologs of the mouse genes will “show up” in the future. Otherwise, it is possible that one or several paralogs compensate the biological function of an “absent” gene. This may be the case for the two mouse paralogs *Shcbp1l*/*Shcbp1:* only *Shcbp1* exhibits one ortholog in chicken and zebrafish, and this ortholog may be sufficient to ensure the biological function in the testes of chickens and zebrafish, while two genes would be necessary in mice.

### Testis expression of conserved genes

We found that the majority of orthologs of the mouse testis-enriched genes are also predominantly expressed in chicken testes (61%). In the zebrafish, almost half of them (46%) have a relative testis expression ≥50%. Conversely, in the fruit fly, among the 28 genes that give rise to the 68 mouse orthologs of interest, only 13 exhibit testis-enriched expression (Additional file 3: Table S3A column F). The 15 remaining genes are expressed in the testis, albeit not preferentially. Twelve (*bel, alphaTub84B, Ance, pAbp, sqd, tra2, tud, Sas-4, Rho1, mip120, dsx, Mhc*) are ubiquitous in the fly, whereas their mouse orthologs are mostly (TE ≥ 50%) expressed in the mouse testes.

Concerning the expression data in chickens, we experimentally investigated a set of 41 (Additional file 3: Table S3A and Additional file 7: Table S5) unique genes or groups of paralogs. For most results, we verified the degree of concordance with available data in Ensembl Expression Atlas, based on the transcriptomic work of Merkin et al. (2012). Quantitative variations between our results and those of Merkin et al. can be observed for some of the genes, such as lower relative testicular expression in our study (Tesmin, 65% in our study versus 100% in Atlas; ENSGAL00000011243, 74.5% in our study versus 99.7% in Atlas). Two principal reasons can explain this apparent discrepancy. First, the number of tissues is not exactly the same between the studies, and second, the chicken lines are different. The chicken line used in the present study was an agronomic broiler, in which most of the genes had a substantial expression in the muscle.

For the zebrafish, one can note that a high relative expression in the ovary can be found for some genes mainly expressed in the testes. This was also true for the mouse species. For example, and as described in our previous work [15], *tdrd5* and *rnf17* are highly expressed in the zebrafish testis and ovary, similar to their mouse orthologs. This finding suggests a common function in male and female germ cells for these conserved genes across evolution. Further investigations on hen ovaries would be helpful to ascertain this point.

### Comparison among mouse/chicken/zebrafish

Among the 625 mouse genes, we selected **68** genes mainly expressed in testes (orthologs of 28 fly genes): **42** had a relative testicular expression level greater than 20% (cut-off arbitrarily chosen) and **26** were paralogs. Among the 42 genes with a relative testicular expression greater than 20%, 34 mouse genes had a relative testicular expression rate higher than 50% and eight genes between 20 and 50%, albeit restricted to a limited number of tissues. Interestingly, among these eight genes with lower relative testicular expression, p*kd2l1* exhibited a chicken ortholog (*PKD2L1*) restricted to testes (TE = 100%) and two zebrafish co-orthologs (*pkd2l1/pkd2*) mainly expressed in the testes. With the same idea, among the 26 paralogs and in spite of their low testicular level in mice, six genes (*Kpna2, Cenpj, Lin54, Ccna2, Shcbp1, Pkd2 –* paralog of *Pkd2l1-*) have chicken and/or zebrafish orthologs with a relative testicular expression levels greater than 50%. For example, the relative expression level of *Cenpj* in mouse testes was less than 9%, while it reached 85.6% in chicken and 63% in zebrafish testes.

We observed numerous duplications for the orthologs of four mouse genes (*Phf7, Sun3, Sun5, Spag4*) in chickens, but not in zebrafish. For example, *Phf7* has 63 predicted co-orthologs in the chicken, but none in the zebrafish. This expanding duplication in numerous paralogs appears to be distinctive to chickens and probably to other avian species, as several paralogs of this gene also exist in zebra finches (38), turkeys (17) and ducks (6) (see tree ENSGT00390000005246 available in Ensembl). This high rate of duplication may correspond to a sub-functionalisation of the various paralogs, as this phenomenon is well-described for paralogs in the testes [for review: [26]] or other tissues, such as fat tissue [27]. Further investigations will help us to answer this question and to develop a greater understanding of bird testicular function specificities.

### Conservation of testicular gene functions

A previous study in 2008 [9] analysed genes predominantly expressed in the testes and conserved between fruit flies and humans. In this study, the authors identified 12 candidate genes putatively involved in male fertility in mammals. The main difference to our present work is that this previous study focused on the genes whose expressions were restricted to the testes in flies and then tried to identify the human orthologs. In contrast, in the present study, we first identified genes whose mutation leads to functional impact (mutation with phenotypical consequence) in the fly, even if the expression of the gene is not restricted to the fly testes, and we then identified the vertebrate orthologs and examined their expression in the testes. Intriguingly, no common gene exists for these two lists of genes reported (their list of 12 conserved genes and our 68 conserved genes). Most likely, this is due to evolution/changes between the time of their (2008) and our study in the content of data in phylogenetic databases, as we have observed for a proportion of our own data (see “Limitations” paragraph in the Discussion). However, and interestingly, both sets of genes include genes involved in spermatozoa structure and nucleic acid metabolism, as described below.

The Gene Ontology term analysis demonstrated a functional similarity between the fruit fly and the mouse, with common GO terms related to germ cell structure and meiosis. In the zebrafish, data gained from morpholino-injected embryos showed defects related to germplasm structure. In the fruit fly, almost all of the 28 genes present a defect in one or several germ cell types when mutated, independent of their testicular expression profile. Indeed, more than half of the 28 genes exhibited a ubiquitous expression pattern, whereas at least one of their mouse co-orthologs was mainly expressed in the testes. Thus, for these genes, the fly mutant phenotype may be the result of direct and indirect effects.

In the mouse, most genes are known to be expressed in male germ cells, with some additionally being expressed in the oocyte (*Dazl, Tdrd1/5/9*). The GO term analysis revealed functions related to nucleic acid binding. Regarding this species, a large body of literature (especially concerning targeted invalidation) provides functional information for about one third of the 68 mouse genes of testis interest (see below). These genes can be classified into four main biological functions, as listed below:

Meiosis process: ***Mtl5*** is expressed in most stages of meiotic prophase I, with a strong expression in late pachytenes [28]. Furthermore, ***D1Pas1*-**deficient male mice are sterile, with spermatogenic arrest at the late pachytene stage [29]. ***CcnA1*** invalidation leads to sterile male mice because of meiosis arrest, followed by germ cell apoptosis [30]. It is not surprising that genes with such functions are highly conserved across evolution, as previous studies have shown that this is true for numerous loci involved in meiotic recombination [31] or checkpoints required for the meiotic cell cycle [32].

Structure of spermatid/spermatozoon: ***Klhl10*** is involved in the spermatid elongation process, and its deficiency leads to sterility [33]. ***Sun3/Spag4*** are proteins involved in germ line nucleus association, at the site of attachment of the single spermatid centriole [34–36]; ***Sun5***-null spermatozoa are acephalic, as Sun5 functions to anchor the sperm head to the tail. In ***Lrrc6−/−*** mice [37], the outer dynein arms are absent from the spermatozoa cilia, leading to incomplete motility. In humans, loss-of-function if this gene is characterised by the absence of dynein-arms [38]. ***Zmynd10*** is important for cilia motility and also interacts with *Lrrc6* for dynein arm assembly [39, 40]. ***Ttll8*** is involved in tubulin glycylation in sperm flagella [41, 42]. ***Armc3/4*** encode members of the superfamily of armadillo repeat proteins, the archetypal modular-binding proteins involved in various fundamental cellular processes, including cell–cell adhesion, cytoskeletal organisation, nuclear import and molecular signalling [43]. ***Armc4*** is required for spermatid maturation [44], and a specific natural mutation in the ***Armc3*** gene has been shown to lead to a sterilising tail sperm defect in bovine species [45].

RNA processing, binding or transport: genes with such functions have been highlighted in our previous study focusing on the evolution of oocyte genes [15]. For example, ***Rnf17*** encodes a protein involved in ribonucleoprotein granules for RNA processing [46, 47]. Germinal granules are strongly implicated in the transport, storage, localisation, stability and regulation of the translation of mRNA in the testes [48, 49]. Other genes involved in the process are well-described, including ***Boll*,** ***Dazl*** and ***Tdrd 5/1/6/9*** [49–53]. Another gene involved in this functional category is ***Papbc2,*** an RNA-binding protein that plays a role in the translational regulation of the mRNAs required in the later stages of spermatogenesis [54]. Its paralog ***Papbc6*** is tagged with “RNA binding” GO, but has no known phenotypical function in the literature.

Interaction with DNA: this function is represented by two genes, ***Phf7***, a protein that binds histone H3 N-terminal tails in humans and flies [55, 56], and ***Dmrtb1*,** known as Dmrt6, which represses the genes involved in spermatogonial differentiation and activates genes required for the meiotic prophase [57].

The GO enrichment analysis confirmed the involvement of genes involved in RNA metabolism and/or transport, with a high degree of enrichment of these GO terms in the list of 68 mouse genes. Functional studies are required to ascertain whether this is also the case in chickens. Note that the chicken ortholog of *Pkd2l1* (*PKD2L1*) is exclusively expressed in the testes, suggesting that this gene is important for chicken spermatogenesis.

### Limitations: false negative genes, no ortholog found, frequent changes in databases

As mentioned in our previous study on genes involved in oogenesis [15], one limitation of our method is that the stringency of the available tools in databases (Ensembl trees and Biomart tools) could not allow us, in some cases, to find an ortholog, even when we knew that the vertebrate ortholog did exist: this was the case for oocyte genes encoding *mos, Vasa and brca2* [15]. It is a bias inherent in meta-analyses on several hundred genes: databases evolve so rapidly that regularly, some phylogenetic trees change their size and the number of their members. We observed discrepancies between Ensembl release 88, when we started the work (2017), and release 96 (2019) at the time of reviewing, especially when we examined the trees and superTREES (available only since October 2018). These discrepancies are mainly due to recent changes in the methods for calculating the gene trees (communication of Ensembl team). Changes are frequent in databases (in the last 5 years, Ensembl data have been updated every 3 months on average), and it is illusory to expect 100% accuracy in orthology links. Thus, our data are not perfect, and we observed both qualitative and quantitative variations. For example: Biomart 96 claimed that 10 fly genes (*bel, pAbp, Pen, sqd, Spag4, dsx, nesd, Mhc, Lam, Abd-B*) do not have any mouse orthologs. However, eight of them belong to the same tree (*Pen*) or superTREE (*bel, pAbp, sqd, nesd, Mhc, Lam, Abd-B*) as the mouse co-orthologs designated in 2017. This observation points out the higher stringency of the Biomart tool versus trees (communication of Ensembl team). Finally, two fly genes become ambiguous: *dsx* and *Spag4.* Indeed, the mouse co-orthologs designated in 2017 exhibit a low Flybase orthology score (4/15 for *dsx* and 5/15 for *Spag4*), whereas the scores of the eight other genes are higher (≥ 10/15). This indicates that crossing information from various databases is useful to consolidate orthology links, especially with species with a large evolutive distance. In particular and interestingly, the mouse orthologs of both *dsx* and *Spag4*, obtained with Biomart 88, exhibit chicken and zebrafish orthologs with a relative testis expression ≥50% (see Additional file 3: Table S3A). Moreover, in any case, if there are false-negatives in our study, the large majority of the orthology links that we observed are true; and if there are false-positives, there are likely very few.

Finally, our analyses are dependent on 1) statistical models that evolve with the massive production of new genomics data from novel species; 2) calculating method changes with time in databases. Moreover, for the same reason of evolutionary distance, the conserved synteny could not be used between *Drosophila* and vertebrates to complete or strengthen our phylogenetic trees. It is therefore possible that, concerning the supposed genes present in the fly but not in the vertebrates, due to the evolutionary distance between these species, these genes may have a true vertebrate ortholog, but that the high stringency of the phylogenetic trees recovered from Ensembl database does not allow to identify them without any ambiguity (example: the *Drosophila* genes *piwi* and *aub* exhibited no vertebrate ortholog at the time of our analysis with Biomart in Ensembl release 88).

## Conclusions

Through an evolutional approach combining the exploitation of various in silico data, we identified new conserved genes predominantly expressed in the testes and required for male reproduction in vertebrates. Indeed, the present study highlights at least three mouse candidate genes that would deserve further functional studies: *Lrrc46, Pabpc6* and *Pkd2l1*. Their relative expression levels in the testes are high in mice, chickens and zebrafish, but no functional data are available in vertebrates. Further studies would help to determine whether these genes have an important function in the testes. Finally, in the case of a species of agronomic interest, the chicken, a substantial list of genes putatively important for male reproduction has emerged, exhibiting high relative expression levels in the testis and being (co)-orthologs of genes important for male fertility in at least one of the other two model species studied.

## Methods

Figure 1 summarises the project workflow, combining in silico (section 1) and experimental data (section 2) and comprising five main steps, as described below and in the results.

### Data retrieval for in silico approach

The data bank interrogations for this study were mostly performed in April 2017 with Ensembl release V88 (accessible in the archive site at https://www.ensembl.org/info/website/archives/index.html: Biomart and phylogenetic trees), and additional recent data (superTREE) were retrieved for our candidate genes from Ensembl release 96 and from Flybase Dmel 6.26 (version 2019_02) (phylogenetic score). Details are presented in each paragraph below.

#### Genes involved in reproductive phenotype in fruit flies (*Drosophila melanogaster*)

The genes involved in *Drosophila melanogaster* male reproduction at the testis level were retrieved using Flybase data (http://flybase.org/, Dmel Release 6.13), a database that collates mutants with specific phenotypes. The database was interrogated with the following keywords: 1) *Phenotypic class* interrogation: “sterile male” and “semi-sterile male”; 2) *Tissues/cell-affected* interrogation: “testis” and “sperm”. Having eliminated redundancy, a list of 379 unique fruit fly genes (Fig. 1 step 1) was established for further analyses. These genes are numbered and listed in Additional file 1: Table S1 (“fruit fly” columns) along with their paralogs, while indicating whether these paralogs belong to the initial list (i.e. if they are additionally involved in testis function) in order to avoid redundancy (in case these paralogs share mouse co-orthologs).

#### Retrieval of vertebrate and invertebrate orthologs

To retrieve the vertebrate (chicken, zebrafish, lizard) orthologs of the fruit fly genes, we used the Biomart tool (http://www.ensembl.org/biomart/martview/5c45d7b6573c2922f8ffecfbb15679f0) of the Ensembl database (last interrogation Ensembl release 88). We also retrieved information in superTREES, which regrouped smaller phylogenetic trees (since Ensembl release 96). Briefly, for the use of the Biomart tool, the “dataset” corresponded to the *Drosophila melanogaster* genes, the “filter” to the 379 ID genes that were copied as “input external references ID list”. In the “Attributes” tab, we checked “homologues” and then each vertebrate “Orthologues” of interest. With these parameters, the results for each fly gene gave the names and IDs of orthologues of all requested species.

In chicken and zebrafish, the orthology relationship was confirmed by a “vetting process”, which consists of tree analyses. In most of the cases, the tree links and the Biomart results were identical. However, discrepancies were not uncommon: we observed that Biomart is sometimes more stringent than the information given by the trees. Thus, when the gene was predicted absent in Biomart, we analysed the Ensembl tree. If the gene was present in the tree (ambiguous cases), we analysed the conservation of syntenic chromosomal fragments using the Genomicus tool (http://www.genomicus.biologie.ens.fr/) or directly accessible for each gene in an Ensembl tree) or the Genome Data Viewer (https://www.ncbi.nlm.nih.gov/genome/gdv/) when no data was available in Ensembl. In both chicken and zebrafish, tBLASTn interrogation with reciprocal verification using the best hits (higher score and lower e-value as a new query) was used when ambiguities occurred, as we have previously described [15]. Moreover, in zebrafish, some trees were built using the Molecular Evolutionary Genetics Analysis (MEGA) software version 7.0, with homolog proteins aligned using the BioEdit ClustalW multiple alignment editor software version 7.1.3.0 (http://www.mbio.ncsu.edu/BioEdit/bioedit.html). Trees were constructed using the Neighbour-Joining method, and the reliability of the inferred trees was assessed using the bootstrap procedure with 1000 replications. For genes lacking vertebrate orthologs, we performed supplemental analyses with EnsemblMetazoa (Biomart and trees: http://metazoa.ensembl.org/biomart/martview/183d7c4a3df65cb3c36c3c905c40e64b, Ensembl release 88) to obtain an overview of the evolution of these genes inside the invertebrate class (Fig. 1 step 2).

#### Expression profile of mouse (*Mus musculus*) genes

Among the mouse orthologs obtained in step 2, we aimed to select genes of interest due to their relative expression in testes, designated as rTE (Fig. 1 step 3). As reported in our previous work [15], we used the Unigene expression profile (http://www.ncbi.nlm.nih.gov/unigene). This database has proved highly reliable in predicting the expression profiles of reproductive genes in mice ([16] [17] [18] [19]). The Unigene expression profile provides the ESTs of 47 tissues (including 45 adult tissues) generated from almost 740 various cDNA libraries and expressed in transcripts per million (TPM). Libraries containing more than 1000 ESTs were considered. For each mouse co-ortholog of fly genes, we retrieved the direct EST profile available in the Unigene page for each gene, as we did previously for ovarian genes [15]. Subsequently, we calculated the total tissular TPM (total expression of one gene in the various murine organs), excluding female tissues (vagina, uterus, ovary, mammary gland, fertilised ovum and oviduct) and embryonic/extra-embryonic tissues, so that the ratio of testis expression was calculated for a male organism as the ratio of male tissues (testes, epididymis, prostate and vesicular glands) and the total tissular TPM. According to the high number of tissues available in Unigene, we retained 42 genes with an rTE ≥ 50%, or between 20 and 50% if expressed in less than five tissues. These cut-offs were arbitrarily chosen. We consider that these genes present a predominant testicular expression and thus are of putative interest for testis function in mice.

#### Orthology link score provided by Flybase

To enrich the list of genes of interest for testis function in mice, we focused on the paralogs of the genes listed above (§1.3) and detected those (26 paralogs) of phylogenetic interest according to the Flybase “Orthologs” tab that summarises the phylogeny links from 15 databases in Flybase Dmel Release 6.26 (Compara, eggNOG, Hieranoid, Homologene, Inparanoid, Isobase, OMA, OrthoDB, OrthoFinder, OrthoInspector, orthoMCL, Panther, Phylome, RoundUp, TreeFam and ZFIN); these orthology links are reflected by a global score (maximum 15) that was retrieved to complete Additional file 3: Table S3A (column J).

#### Expression profiles of fruit fly genes and of chicken (*Gallus gallus*) and zebrafish (*Danio rerio*) orthologs

Gene expressional data were obtained from the following databases: for **fruit fly** genes, data were obtained in Flybase with the tab “Expression data” (sub-tag: “High-Throughput Expression Data/modENCODE Anatomy RNA-seq”); for **chicken** orthologs, our experimental data (see paragraph “Experimental data” below) were confirmed with data in the “Gene expression” display of Ensembl, with these data being based on the work of Merkin et al. [58]. For **zebrafish** orthologs, in silico expression data were obtained in the PhyloFish database [59] (Fig. 1 step 5). In contrast to mouse genes, the threshold for designating a predominant expressed gene in testes corresponded to rTE ≥ 50% (versus 20% in mice) in chicken and zebrafish, in view of the lower tissue number investigated (8 and 10, respectively, in chicken and zebrafish versus 45 in mice). The tissues in chicken are described below (“chicken biological samples”). Ten different tissues were selected in zebrafish: brain, muscle, liver, head kidney, gills, bones, intestine, heart, testis and ovary.

#### In silico functional data of mouse and zebrafish genes

Mutant phenotypes were retrieved (when available) from the following data banks: Flybase for fly orthologs (“Summary of Phenotypes” tab), Mouse Genome Informatics (MGI: http://www.informatics.jax.org/; “Phenotypes&Mutant Alleles” tab) for mouse orthologs, Zfin (https://zfin.org/) and literature review for zebrafish orthologs. In the case of the flies and mice, only the mutant exhibiting the most deleterious reproductive outcome is indicated for each gene.

#### Gene ontology (GO) of mouse and fly genes

“Biological Process” and “Molecular Function” Gene Ontology (GO) were investigated using the DAVID database (Functional Annotation Tool: https://david.ncifcrf.gov/summary.jsp) or the Biomart tool of Ensembl.

The DAVID database was used to determine enrichment compared with the whole genome in flies. Briefly, the 379 ID fly genes were pasted in the “Functional Annotation Tool” as a “gene list” and submitted using two parameters: “GOTERM_MF_DIRECT” and “GOTERM_BP_DIRECT” (other parameters were unchecked). The data were obtained as a chart including Fold Enrichment and adjusted *P*-Value data (Bonferroni test), directly downloaded as the Additional file 6: Table S4.

The Biomart tool of the Ensembl database was used to compare the GOs between two lists of genes in mice. After copying a gene list in “Input external references ID” in the “Filters” tab, the parameters chosen in “Attributes” were: “Features”, then Gene stable ID in “GENE”, then GO term accession and name in “EXTERNAL”. The result of such interrogation corresponded to all GOs linked to the gene list. The emergence frequency of a specific GO in the two lists investigated was compared via a Chi-square test.

### Experimental data of gene expression in mice and chickens

#### Mouse and chicken biological samples

Animals were provided by INRA (Institut National de la Recherche Agronomique) local farm facilities, which are officially authorised by the French Ministry of Agriculture for breeding and animal experimentation (for mice: farm facility is UE-PAO, agreement number E-37-175-2; for chickens: farm facility is UE-PEAT, agreement number D-37-175-1). Mice (Swiss strain locally breeded) and chickens (Leghorn line locally breeded) were raised in their respective animal husbandries with standard breeding conditions (diet, temperature and L: D photoperiod), and handling and sacrifice protocols were approved by the French Ministry of Agriculture and the local official ethics committee (Comité d’Ethique en Expérimentation Animale Val de Loire CEEA – n°19), in accordance with the European Directive 2010/63/EU on the protection of animals used for scientific purposes. All efforts were made to minimise animal stress. Animals necessary for the study were euthanized: mice (adult, 2.5-months-old; *N* = 6 males) were euthanized by cervical dislocation, and unconscious (after electrical stunning) chickens (adult, 8-months-old; *N* = 3 males) were bled. The tissues sampled for mice comprised testis (T), epididymis (Ep), muscle (M), liver (L), spleen (S), brain (B), kidney (K) and heart (H). For chickens, they included testis (T), vas deferens (VD), brain (B), muscle (M), lung (Lu), heart (H), liver (Li) and intestine (I). The tissues were sampled rapidly and immediately frozen in liquid nitrogen to be kept at − 80 °C until RNA extraction.

#### RNA extraction, reverse transcription and quantitative PCR for mouse and chicken genes

Total RNA was isolated from frozen tissues using RNAble reagent (Eurobio) according to the manufacturer’s instructions. The RNA (1 μg) was reverse-transcribed after DNAse I treatment (Promega), using RT-MMLV from Promega and oligo (dT) (Promega) primers according to the manufacturer’s instructions. The PCR reaction included two negative controls: water and RNA samples not retro-transcribed (RT-). Real-time PCR was carried out in triplicates with SYBR Green reagent (BioRad), in accordance with the manufacturer’s instructions, in a final volume of 20 μl. Primers (Additional file 7: Table S5) were designed with the NCBI “primer-blast” tool, so that primer pairs had a 60 °C annealing temperature. In case of numerous paralogs and due to their high percentage of sequence identity, it was impossible to design primers specific to each paralog: consensus sequences between paralogs were found with the MultAlin tool available at http://multalin.toulouse.inra.fr/multalin/, so that the primers amplify groups of several paralogs. Fluorescence was detected on a MyiQ™ cycler (BioRad, Marnes La Coquette, France) with the following conditions: 35 cycles with denaturation at 95 °C for 30 s, specific annealing at 60 °C for 30 s and elongation at 72 °C for 30 s. The specificity of amplified fragments was controlled by checking the existence of a single peak on the melting curve and by verifying their sequence (amplicon sequencing services of Genewiz: www.genewiz.com/en-GB/). We checked that each set of primers had an efficiency comprised between 80 and 120%. For normalisation, the internal standard (housekeeping gene), exhibiting a similar expression in the various tissues, was *Rpl19* in mice (sense primer: CCTCCAGGCCAAGAAGGAAG; anti-sense primer GGGCAACAGACAAAGGCTTG). In chickens, we tested two housekeeping genes: *EEF1A* (eukaryotic translation elongation factor 1 alpha 1) (sense primer: AGCAGACTTTGTGACCTTGCC; anti-sense primer: TGACATGAGACAGACGGTTGC) and *RPL15* (ribosomal protein L15) (sense primer: TGTGATGCGTTTCCTCCTTGG; anti-sense primer: CCATAGGTTGCACCTTTTGGG). Given that they exhibited similar expression profiles, normalisation was performed with *EEF1A* level for graphical representation. For each gene, tissular expression is presented as a relative mRNA level proportionally to testis level (with the arbitrary level being 100).

### Statistical analysis

Chi-square test analyses were performed to explore the proportions of genes conserved in invertebrates and vertebrates, as well as the enriched GO in mice. When necessary, ANOVA, followed by Bonferroni *post-hoc* tests, was applied to compare tissular expression in chickens.

## Additional files

Additional file 1: **Table S1.** Columns B to I: fly genes List of the 379 Drosophila genes with male reproductive phenotype when mutated (A-F) and their paralogs (G-I). Columns J to BL: mouse genes. The mouse orthologs of the 379 fly genes are listed (columns J-M) and for each of them their expression in adult mice organs (according to Unigene EST profile) is provided (for calculation of their relative testis/ovary expression: columns BG-BL). (XLSX 691 kb)

Additional file 2: **Table S2.** FLY genes without ortholog in chordates. These genes are extracted from (Additional file 1: Table S1) for more readability. In blue: genes having a tree in Ensembl (release 88) In orange: genes with no tree in Ensembl (release 88) but in EnsemblMetazoa (release 88). (XLSX 42 kb)

Additional file 3: **Table S3A.** List of the 28 fly genes (columns B-F), whose mouse orthologs (68) are enriched in testis (G-L). Chicken (M-R) and zebrafish (S-X) orthologs were retrieved with Biomart tool, and their syntenic position and tissular expression were analysed. Mutant phenotype was indicated in fly (E), and when available in mouse (L) and zebrafish (X). **Table S3B.** Lines 1 to 11: mouse/chicken orthology links of the testicular genes described in Table S3A Lines 12 to 21: mouse/zebrafish orthology links of the testicular genes described in Table S3A. (ZIP 50 kb)

Additional file 4: **Figure S1.** relative mRNA expression level in mouse tissues (T: testis; M: muscle; Ep: epididymis; Li: liver; S: spleen; B: brain; K: kidney; H: heart. N=6 adult males) determined by qRT-PCR for 20 genes (see primers supplemental Table 5) among the 68 of interest (Additional file 3: Table S3A). The testis level is arbitrarily equal to 100. Normalisation was performed with Rpl19 housekeeping mRNA level. (PDF 44 kb)

Additional file 5: **Figure S2.** relative mRNA expression level in chicken tissues (T: testis; I: intestine; Li: liver; Lu: lung; VD: Vas Deferens; M: muscle; B: brain; H: heart. N=3 different adult males) determined by qRT-PCR for genes of interest in chickens (see primers in Additional file 7: Table S5). These results are described in Additional file 3: Table S3A. A: genes with relative expression in testis ≥50% (enriched) B: genes with relative expression in testis between 20-50% C: genes with relative expression in testis <20% (not enriched) The testis level is arbitrarily equal to 100. Normalisation was performed with EEF1A housekeeping genes. Indicated for each gene: Ensembl name (or mice ortholog for “novel gene”) and Ensembl ID, with the exception of four sets of mouse Sun3, Sun5/Spag4, Phf7 co-orthologs: Sun3 co-orthologs (11 “novel genes”): ENSGALG00000033219 ENSGALG00000043899 ENSGALG00000045507 ENSGALG00000040197 ENSGALG00000040775 ENSGALG00000041021 ENSGALG00000037121 ENSGALG00000046353 ENSGALG00000037958 ENSGALG00000013105 ENSGALG00000038308 Sun5/Spag4 co-orthologs (17 “novel genes”): ENSGALG00000044320 ENSGALG00000044356 ENSGALG00000044446 ENSGALG00000044958 ENSGALG00000045379 ENSGALG00000045598 ENSGALG00000045929 ENSGALG00000045972 ENSGALG00000046099 ENSGALG00000046178 ENSGALG00000046242 ENSGALG00000046257 ENSGALG00000046307 ENSGALG00000046324 ENSGALG00000046493 ENSGALG00000046634 Phf7 co-orthologs (set A 13 “novel genes”): ENSGALG00000044361 ENSGALG00000046514 ENSGALG00000044587 ENSGALG00000044556 ENSGALG00000046230 ENSGALG00000044918 ENSGALG00000044445 ENSGALG00000045579 ENSGALG00000045161 ENSGALG00000046563 ENSGALG00000044499 ENSGALG00000044839 ENSGALG00000044931 Phf7 co-orthologs (set B 13 “novel genes”): ENSGALG00000046190 ENSGALG00000044032 ENSGALG00000044516 ENSGALG00000045973 ENSGALG00000045663 ENSGALG00000045774 ENSGALG00000046608 ENSGALG00000046050 ENSGALG00000046591 ENSGALG00000045432 ENSGALG00000045345 ENSGALG00000046666 ENSGALG00000045008. (PDF 143 kb)

Additional file 6: **Table S4.** List of GOs (BP GOTERM) corresponding to the 379 fly genes according to DAVID Functional Annotation Tool, with Fold Enrichment (column E) and Bonferroni P-value (column F). (XLSX 60 kb)

Additional file 7: **Table S5.** Primer sequences used for expression analysis (qRT-PCR) of the mouse (Additional file 4: Figure S1) and chicken genes (Fig. 3 and Additional file 5: Figure S2) (XLSX 33 kb)

## Abbreviation

BP: Biological process
EST: Expressed sequence tag
GnRH: Gonadotropin-releasing hormone
GO: Gene Ontology
ID: Identity
LH: Luteinizing hormone
MF: Molecular function
rTE: Relative testis expression
TMP: Transcripts per million
