# Supplementary figures and images for: An evolutionary approach to recover genes predominantly expressed in the testes of the zebrafish, chicken and mouse
Source: BMC Evol Biol. 2019 Jul 3;19:137. doi: 10.1186/s12862-019-1462-8 (PMC6609395; doi:10.1186/s12862-019-1462-8)

Fig S1

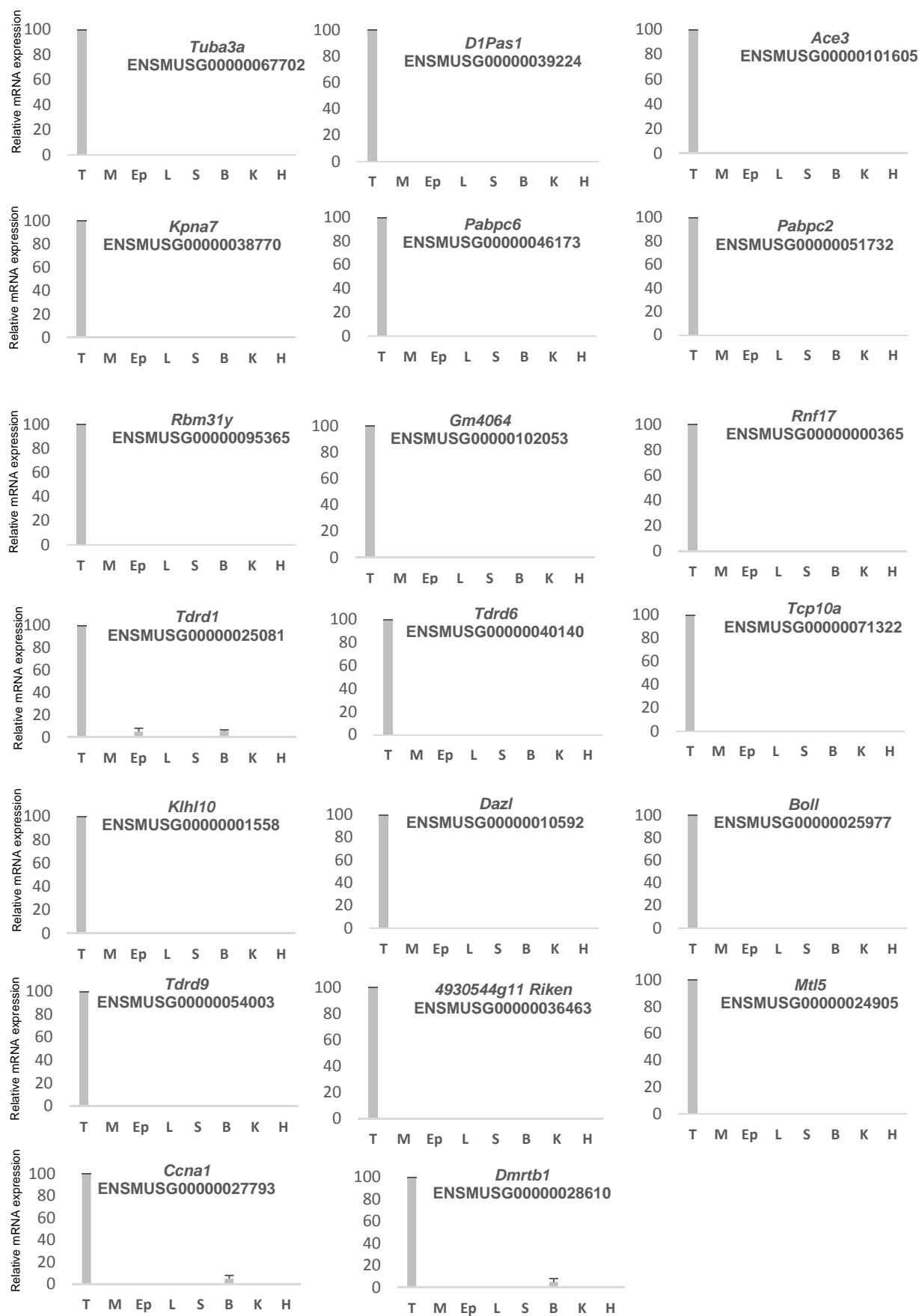

Supplement: Supplementary file 4 — Figure S1. relative mRNA expression level in mouse tissues (T: testis; M: muscle; Ep: epididymis; Li: liver; S: spleen; B: brain; K: kidney; H: heart. N=6 adult males) determined by qRT-PCR for 20 genes (see primers supplemental Table 5) among the 68 of interest (Additional file 3: Table S3A). The testis level is arbitrarily equal to 100. Normalisation was performed with Rpl19 housekeeping mRNA level. (PDF 44 kb) [file 12862_2019_1462_MOESM4_ESM.pdf]
