# Supplementary material for: An evolutionary approach to recover genes predominantly expressed in the testes of the zebrafish, chicken and mouse
Source: BMC Evol Biol. 2019 Jul 3;19:137. doi: 10.1186/s12862-019-1462-8 (PMC6609395; doi:10.1186/s12862-019-1462-8)

Fig S2 (in addition to Fig 3)

A: genes with relative expression in chicken testis ≥ 50%

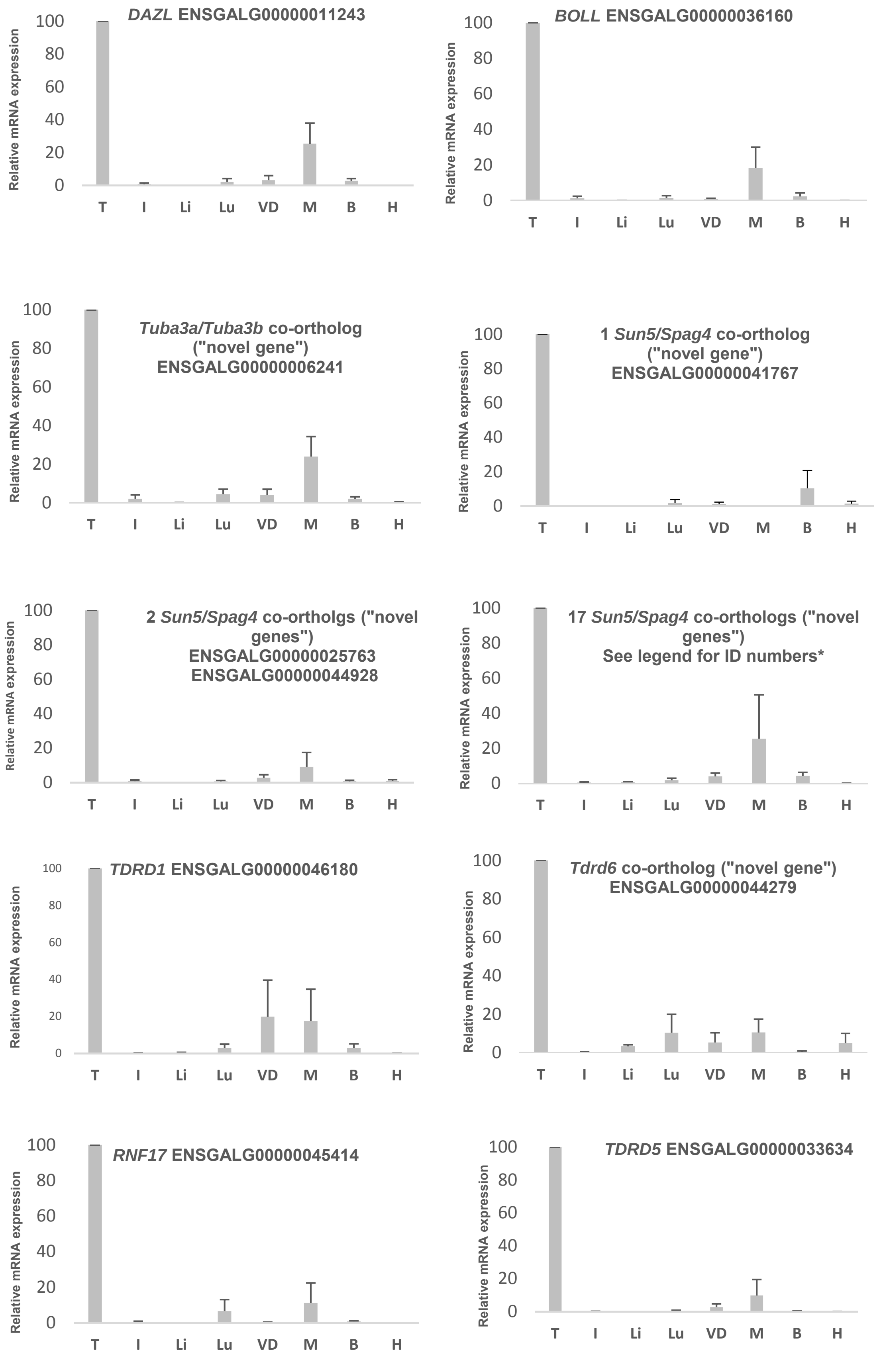

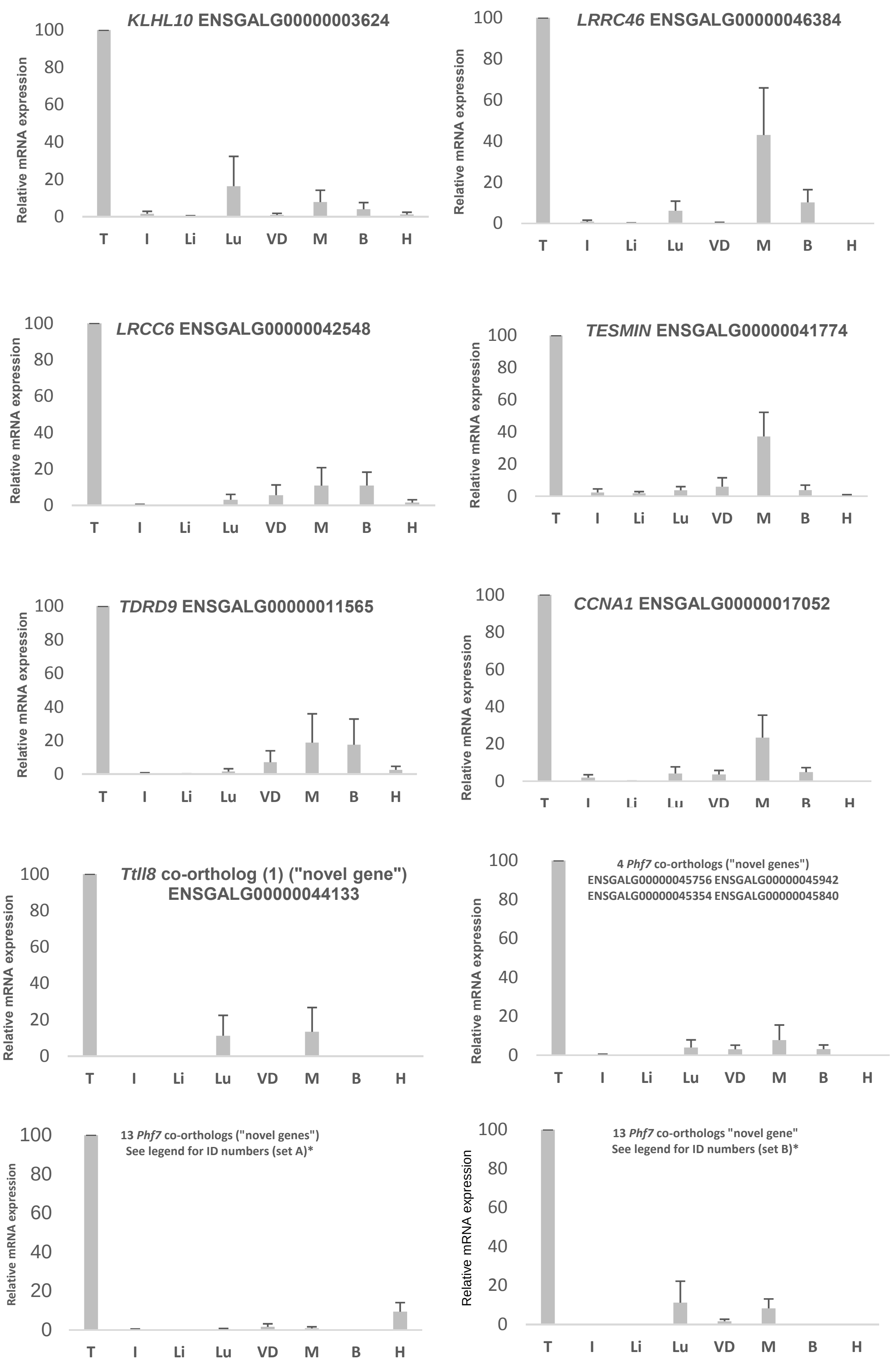
KLHL10 ENSGALG00000003624
LRRC46 ENSGALG00000046384
LRCC6 ENSGALG00000042548
TESMIN ENSGALG00000041774
TDRD9 ENSGALG00000011565
CCNA1 ENSGALG00000017052
Ttll8 co-ortholog (1) ("novel gene")
ENSGALG00000044133
4 Phf7 co-orthologs ("novel genes")
ENSGALG00000045756 ENSGALG00000045942
ENSGALG00000045354 ENSGALG00000045840
13 Phf7 co-orthologs ("novel genes")
See legend for ID numbers (set A)*
13 Phf7 co-orthologs "novel gene"
See legend for ID numbers (set B)*
Relative mRNA expression
0
20
40
60
80
100
T
I
Li
Lu
VD
M
B
H

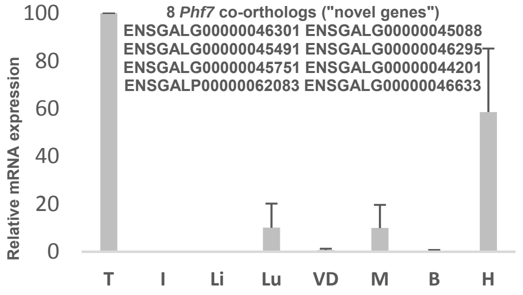

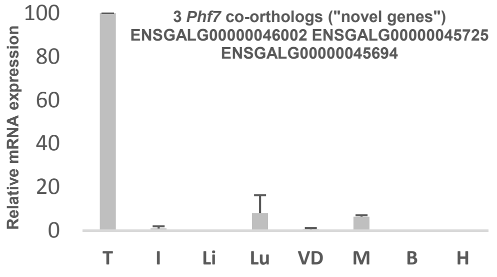

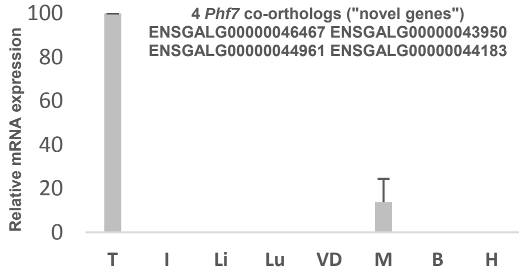

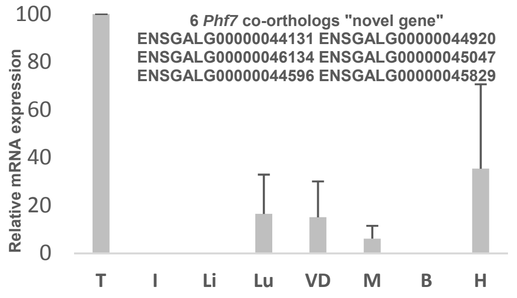

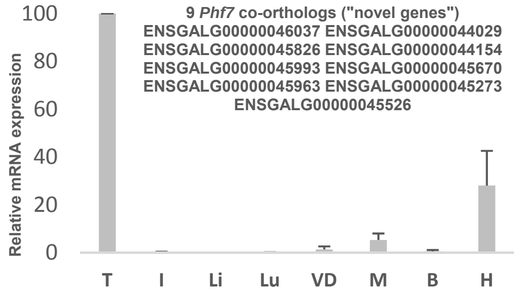

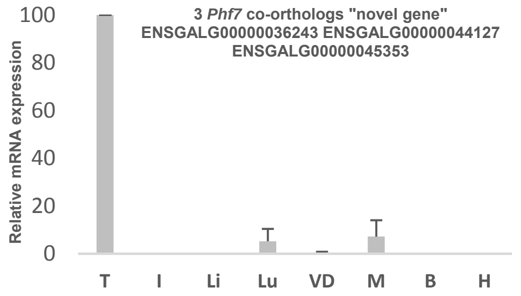

B: genes with relative expression in chicken testis ≥20% and <50%

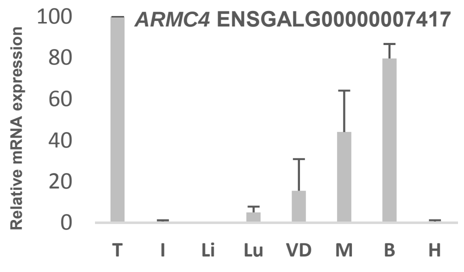

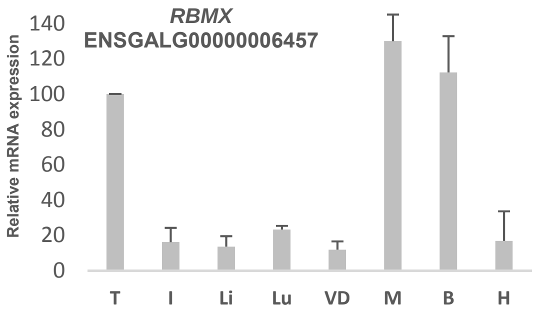

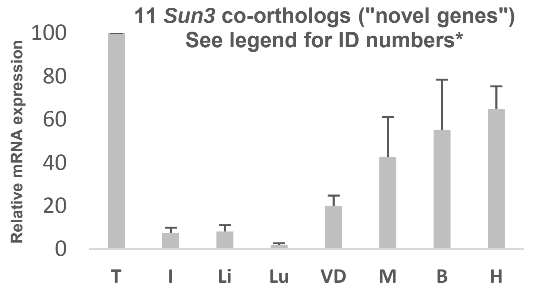

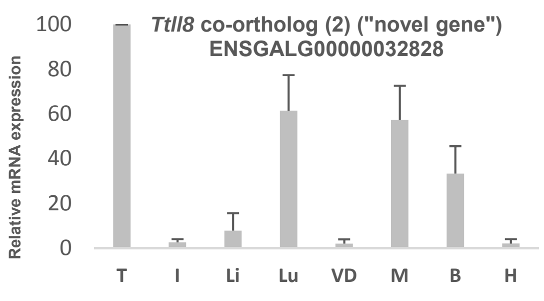

C: genes with relative expression in chicken testis <20%

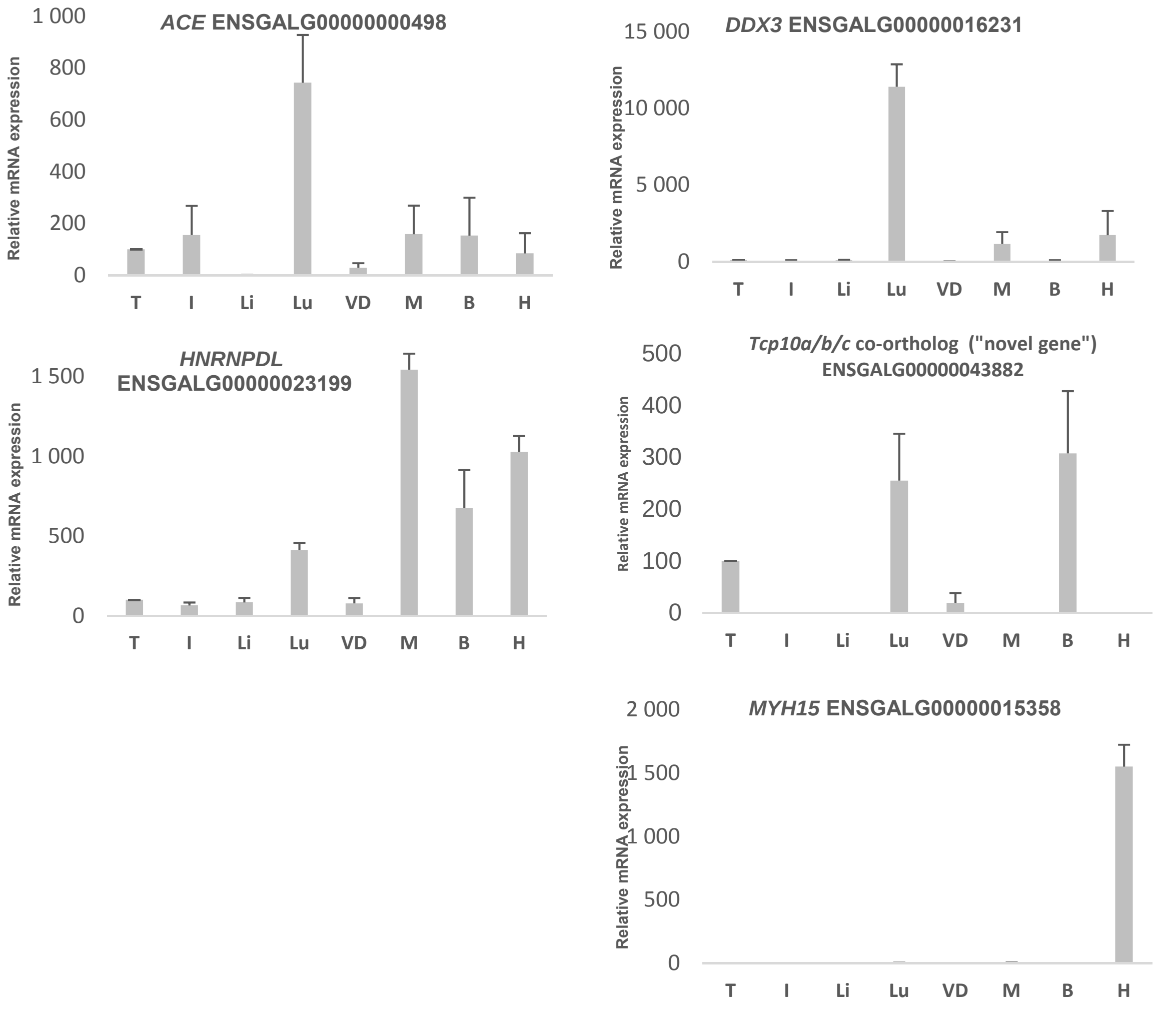

Supplement: Supplementary file 5 — Figure S2. relative mRNA expression level in chicken tissues (T: testis; I: intestine; Li: liver; Lu: lung; VD: Vas Deferens; M: muscle; B: brain; H: heart. N=3 different adult males) determined by qRT-PCR for genes of interest in chickens (see primers in Additional file 7: Table S5). These results are described in Additional file 3: Table S3A. A: genes with relative expression in testis ≥50% (enriched) B: genes with relative expression in testis between 20-50% C: genes with relative expression in testis <20% (not enriched) The testis level is arbitrarily equal to 100. Normalisation was performed with EEF1A housekeeping genes. Indicated for each gene: Ensembl name (or mice ortholog for “novel gene”) and Ensembl ID, with the exception of four sets of mouse Sun3, Sun5/Spag4, Phf7 co-orthologs: Sun3 co-orthologs (11 “novel genes”): ENSGALG00000033219 ENSGALG00000043899 ENSGALG00000045507 ENSGALG00000040197 ENSGALG00000040775 ENSGALG00000041021 ENSGALG00000037121 ENSGALG00000046353 ENSGALG00000037958 ENSGALG00000013105 ENSGALG00000038308 Sun5/Spag4 co-orthologs (17 “novel genes”): ENSGALG00000044320 ENSGALG00000044356 ENSGALG00000044446 ENSGALG00000044958 ENSGALG00000045379 ENSGALG00000045598 ENSGALG00000045929 ENSGALG00000045972 ENSGALG00000046099 ENSGALG00000046178 ENSGALG00000046242 ENSGALG00000046257 ENSGALG00000046307 ENSGALG00000046324 ENSGALG00000046493 ENSGALG00000046634 Phf7 co-orthologs (set A 13 “novel genes”): ENSGALG00000044361 ENSGALG00000046514 ENSGALG00000044587 ENSGALG00000044556 ENSGALG00000046230 ENSGALG00000044918 ENSGALG00000044445 ENSGALG00000045579 ENSGALG00000045161 ENSGALG00000046563 ENSGALG00000044499 ENSGALG00000044839 ENSGALG00000044931 Phf7 co-orthologs (set B 13 “novel genes”): ENSGALG00000046190 ENSGALG00000044032 ENSGALG00000044516 ENSGALG00000045973 ENSGALG00000045663 ENSGALG00000045774 ENSGALG00000046608 ENSGALG00000046050 ENSGALG00000046591 ENSGALG00000045432 ENSGALG00000045345 ENSGALG00000046666 ENSGALG00000045008. (PDF 143 kb) [file 12862_2019_1462_MOESM5_ESM.pdf]
